# The effect of sacubitril/valsartan on urinary C-peptide excretion and endogenous insulin secretory capacity in a patient with type 2 diabetes: a case report

**DOI:** 10.1186/s40780-025-00472-z

**Published:** 2025-08-17

**Authors:** Shun Onodera, Masashi Miyamae, Issei Higuchi, Shunsuke Nashimoto, Akinobu Nakamura, Mitsuru Sugawara, Yoh Takekuma

**Affiliations:** 1 Department of Pharmacy, Hokkaido University Hospital, Sapporo, Hokkaido Japan Japan; 2 Faculty of Pharmaceutical Sciences, Hokkaido University, Sapporo, Hokkaido Japan; 3 Department of Rheumatology, Endocrinology and Nephrology, Faculty of Medicine, Graduate School of Medicine, Hokkaido University, Sapporo, Hokkaido Japan

**Keywords:** C-peptide, Glucagon stimulation test, U-CPR, Urinary C-peptide, Sacubitril/valsartan, ARNI, Neprilysin, Type 2 diabetes, Insulin secretory capacity

## Abstract

**Background:**

The evaluation of endogenous insulin secretory capacity is important in the selection of diabetes treatment. C-peptide, which is secreted in equivalent amounts as insulin, is a versatile test for this evaluation. Urinary C-peptide is widely used because it is less invasive. Sacubitril/valsartan, used to treat hypertension and chronic heart failure, has been reported to increase urinary C-peptide levels; however, its effect on endogenous insulin secretion remains unknown. In this report, we present a case in which insulin secretory capacity was evaluated according to a glucagon stimulation test in addition to urinary C-peptide levels in a patient receiving sacubitril/valsartan.

**Case presentation:**

A male patient in his 50s with type 2 diabetes and hypertension, without renal dysfunction, was treated with sacubitril/valsartan. The results of the glucagon stimulation test showed a C-peptide change of 2.28, and the C-peptide index on the same day was 1.25, indicating normal endogenous insulin secretory capacity. In contrast, 24-h urinary C-peptide excretion was abnormally high at 615.2 µg/day. After discontinuation of sacubitril/valsartan, urinary C-peptide excretion decreased over time (615.2 to 369.0 µg/day), but blood glucose levels did not increase during this period.

**Conclusions:**

In this case, 24-h urinary C-peptide excretion was abnormally elevated despite preserved endogenous insulin secretory capacity, as assessed by the glucagon stimulation test. Although this observation is based on a single case and cannot be generalized, it suggests that sacubitril/valsartan may interfere with the interpretation of urinary C-peptide levels. Therefore, in such clinical contexts, dynamic tests such as the glucagon stimulation test may serve as a useful adjunct to avoid potential overestimation of insulin secretory capacity when relying solely on urinary C-peptide levels.

## Background

The evaluation of endogenous insulin secretion in type 2 diabetes is important for understanding its pathophysiology, guiding treatment decisions, and predicting patient outcomes [1]. Regular evaluation and appropriate treatment according to changes in disease status are expected to provide good glycemic management and prevent complications [1].

The concentration of insulin in the blood cannot be accurately measured to reflect endogenous insulin secretion in patients receiving insulin therapy, as endogenous and exogenous insulin cannot be distinguished. However, C-peptide (CPR) is not present in exogenous insulin and is produced simultaneously with endogenous insulin in equal molar amounts; therefore, it can be used to assess the endogenous insulin secretory capacity even during insulin therapy [2]. CPR is metabolized more slowly than insulin and has a longer half-life in the blood, which reduces variability in measurements [1]. CPR can be measured in both blood and urine, with blood CPR reflecting insulin secretion at the time of blood collection and urine CPR reflecting insulin secretion over time [2].

The assessment of insulin secretion using CPR includes the 24-h urinary C-peptide excretion rate (U-CPR), C-peptide index (CPI), and glucagon stimulation test [2]. U-CPR reflects total daily insulin secretion by measuring urinary C-peptide levels collected over 24 h. The normal range is 60–100 µg/day, with values below 20 µg/day indicating impaired secretion [2]. CPI has been calculated as (fasting serum C-peptide/fasting blood glucose) × 100, with values below 0.8 indicating impaired insulin secretion [2, 3]. The glucagon stimulation test measures changes in serum C-peptide(ΔCPR) before and after intravenous glucagon administration and is used as an indicator of stimulated insulin secretion; a ΔCPR below 1.0 ng/mL indicates insulin dependence [4].

We present the case of a patient with type 2 diabetes who was administered sacubitril/valsartan and exhibited an abnormally high U-CPR despite normal ΔCPR and CPI values. Sacubitril/valsartan is a neprilysin inhibitor and angiotensin II receptor blocker indicated for the treatment of chronic heart failure and hypertension. In this case, we describe the relationship between abnormally high U-CPR and sacubitril/valsartan in the context of a more physiologically dynamic evaluation of endogenous insulin secretion. Unlike previous reports that relied on static measurements such as fasting CPI or serum C-peptide levels [5–7], we employed the glucagon stimulation test to calculate ΔCPR, which provides insight into stimulated β-cell function and insulin reserve. Compared to static indices such as fasting CPI or serum CPR, ΔCPR provides a dynamic assessment of β-cell responsiveness to stimulation [8]. In this case, it helped to clarify the insulin secretory function when the U-CPR appeared disproportionately elevated.

## Case presentation

A male patient in his 50s with type 2 diabetes, hypertension, and hyperlipidemia was admitted to our hospital for the treatment of type 2 diabetes and diabetes education. Serum creatinine was 0.75 mg/dL, eGFR was 84.1 mL/min/1.73 m^2^, and renal function was normal. Before admission, the patient was taking luseogliflozin (2.5 mg/day), alogliptin (25 mg/day), imeglimin (1000 mg/day), and sacubitril/valsartan (200 mg/day). The day of admission was designated as day 1. Alogliptin and imeglimin were discontinued on day 1. On day 2, metformin (500 mg/day) and insulin glargine (4 U/day) were initiated. On day 3, the dose was increased to 8 units/day of insulin glargine, and 8 units/day of insulin glulisine was added. On day 4, the dose was increased to 10 units/day of insulin glargine and 10 units/day of insulin glulisine, and on day 6, the dose was increased to 14 units/day of insulin glargine and 16 units/day of insulin glulisine. On day 7, a glucagon stimulation test was performed, showing a ΔCPR of 2.28 ng/mL and CPI of 1.25, confirming normal endogenous insulin secretory capacity (Table 1). On day 8, tirzepatide (2.5 mg) was administered, and the dose was reduced to 10 units/day for insulin glargine and 4 units/day for insulin glulisine.

**Table 1:** Assessment of insulin secretory capacity on day 7 using the glucagon stimulation test

Item	Pre-glucagon load value	Six-minute post-glucagon load value	Change
Blood glucose(mg/dL)	100	105	5.00
Serum C-peptide (ng/mL)	1.25	3.53	2.28
CPI	1.25	3.36	-

A 24-h urine collection conducted from days 8 to 9 showed an abnormally high urinary C-peptide excretion of 615.2 µg/day. Sacubitril/valsartan was discontinued on day 10. A follow-up 24-h urine collection from days 12 to 13 showed a decrease in U-CPR to 488.5 µg/day. Subsequent collection from days 15 to 16 demonstrated a further decrease, with U-CPR falling to 369.0 µg/day.

**Fig. 1:**
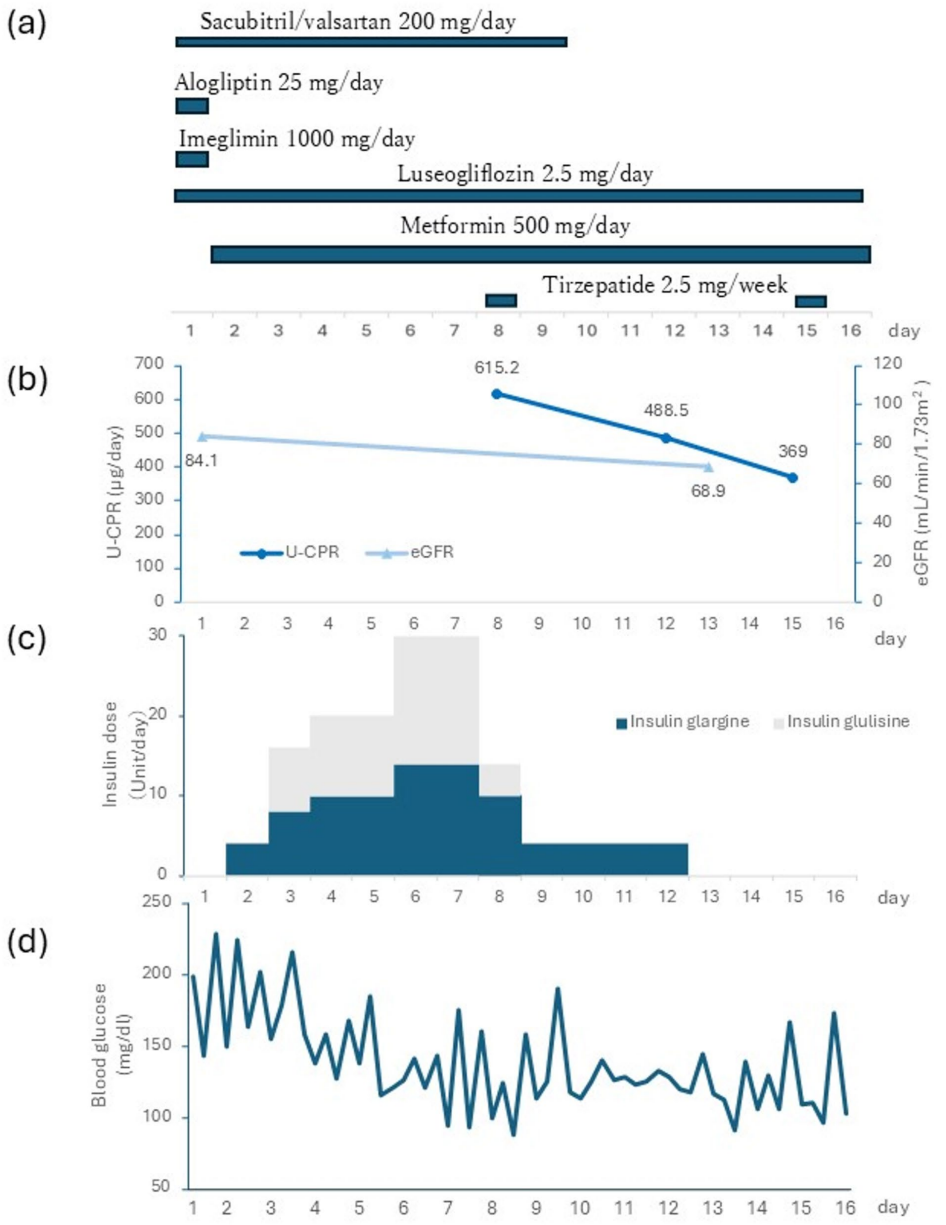
Patient’s clinical course (**a**) Schedule of drug administration, (**b**) changes in U-CPR and eGFR over time, (**c**) total daily insulin shown as a stacked bar chart over 24 h, with insulin glargine and insulin glulisine components, and (**d**) blood glucose levels over time

Initial evaluation using the glucagon stimulation test revealed normal insulin secretory capacity. However, the subsequent 24-h urinary C-peptide excretion was abnormally high, suggesting hypersecretion. This discrepancy highlights a significant inconsistency between the different methods for assessing endogenous insulin secretion capacity. In addition, after the discontinuation of sacubitril/valsartan, a decrease in endogenous insulin secretion was expected to accompany a decrease in U-CPR; however, blood glucose levels did not increase despite the discontinuation of insulin therapy during this period (Fig. 1).

Blood glucose, serum C-peptide concentration, and C-peptide index (CPI) values before (0 min) and 6 min after glucagon loading. The change was calculated by subtracting the pre-glucagon load value from the 6-min post-glucagon load value. Blood glucose and serum C-peptide concentrations were expressed as mg/dL and ng/mL, respectively.

## Discussion and conclusions

In this case, the endogenous insulin secretion capacity was assessed not only by serum C-peptide but also by ΔCPR through a glucagon stimulation test, which more accurately confirmed that the endogenous insulin secretion capacity was normal. While ΔCPR and CPI remained within normal ranges, U-CPR was the only parameter that showed an abnormally high value. Despite this elevation, endogenous insulin secretion was preserved based on the results of the glucagon stimulation test. Interestingly, U-CPR levels gradually decreased after discontinuation of sacubitril/valsartan; however, blood glucose levels remained stable even after insulin therapy was stopped. This suggests that the decrease in U-CPR levels did not reflect a deterioration in endogenous insulin secretion. Instead, stable glycemic control was likely due to the resolution of glucotoxicity achieved through prior insulin therapy.

We reviewed previously reported cases in which the U-CPR levels increased during sacubitril/valsartan therapy [5–7, 9, 10]. Including the present case, Table 2 summarizes six such reports, detailing the number of cases, sacubitril/valsartan dose, changes in U‑CPR, other evaluation methods, and renal function. While many studies have assessed insulin secretory capacity using static indices such as serum C-peptide or CPI, few studies have additionally incorporated dynamic assessments such as the meal tolerance test (MMT). These dynamic approaches may provide supportive and potentially more reliable insights, particularly in situations where U‑CPR alone is difficult to interpret.

Among these methods, the glucagon stimulation test offers certain advantages. Specifically, it is simple to perform, can be completed in a short time, and exhibits good reproducibility [8]. Unlike the MMT, the glucagon stimulation test is less affected by individual variations in gastric emptying or nutrient absorption and allows for standardized, glucose-independent evaluation of pancreatic β-cell responsiveness [8]. Additionally, ΔCPR has been reported to correlate more strongly with pancreatic β-cell mass than do other markers such as fasting serum C-peptide, CPI, or immunoreactive insulin (IRI) [11].

A recent case report by Wang et al. [9] described a patient with type 2 diabetes who exhibited markedly elevated urinary C-peptide levels during sacubitril/valsartan therapy, including a rebound increase after rechallenge. While their report provided valuable observations, the assessment of insulin secretory capacity was based primarily on static markers such as serum CPR and CPI, with the glucagon stimulation test performed only after drug discontinuation. Urinary C-peptide levels in their case were calculated using spot urine samples that may be affected by hydration status or diurnal variation. In contrast, our case incorporated dynamic assessment using the glucagon stimulation test during sacubitril/valsartan administration and consistently used 24-h urine collections for U‑CPR throughout the study period. This methodological consistency strengthens the reliability of our findings and allows for a more physiologically integrated interpretation of insulin secretory capacity under the pharmacological influence of sacubitril/valsartan therapy.

A recent study by Haraguchi et al. highlighted that the glucagon-stimulated ΔCPR was significantly reduced in patients receiving GLP-1 receptor agonists, suggesting that incretin-based therapies may blunt the insulinotropic response to pharmacological stimulation and interfere with the accurate assessment of β-cell function [12]. Accordingly, the glucagon stimulation test may not be appropriate for evaluating the insulin secretory capacity in patients currently receiving GLP-1 receptor agonists. In the present case, the glucagon stimulation test was conducted without concurrent GLP-1 receptor agonist use and renal function was within the normal range. These conditions likely contributed to a more reliable assessment of the endogenous insulin secretory capacity under relatively controlled physiological conditions.

**Table 2:** Summary of published cases reporting urinary C-Peptide elevation during sacubitril/valsartan therapy

Author (Year)	Number of Case(s)	Sacubitril/ Valsartan Dose (mg/day)	U-CPR (µg/day)	Other markers	eGFR (mL/min/1.73m^2^)
Nishiya et al. (2022)	6 cases (show 1 case)	Not reported	1079 to 61*	serum CPR	Not reported (described as > 30 mL/min/1.73 m²)
Tanji et al. (2023)	6 cases (show 1 case)	200	307 to 99	serum CPR	75
Itoh et al. (2024)	1 case	100	400 to 62	CPI	51
Wang et al. (2025)	1 case	50	650 to 86*	CPI, ΔCPR (off ARNI)	73
Kondo et al. (2025)	2 cases (show 1 case)	400	685 to 367	CPI, MMT	68
Present case	1 case	200	615 to 369	CPI, ΔCPR (on ARNI)	83

This table summarizes published case reports in which urinary C-peptide excretion (U-CPR) increased during sacubitril/valsartan treatment. For each study, the number of reported cases, sacubitril/valsartan dose, changes in U-CPR (before and after discontinuation of sacubitril/valsartan), other markers used to evaluate the insulin secretory capacity, and estimated glomerular filtration rate (eGFR) are listed. When multiple cases were presented, only one representative case with detailed information was included. “Not reported” indicates that the information was not provided in the original publication, and “described as > 30 mL/min/1.73 m²” reflects qualitative descriptions without specific values. The term “on ARNI” denotes measurements obtained during therapy with sacubitril/valsartan, a representative angiotensin receptor–neprilysin inhibitor (ARNI), whereas “off ARNI” denotes measurements obtained after discontinuation of the drug. Creatinine-corrected U-CPR values (µg/g·Cr) are marked with an asterisk (*). Abbreviations: CPR = C-peptide immunoreactivity; CPI = C-peptide index; IRI = immunoreactive insulin; MMT = meal tolerance test; ΔCPR = change in serum C-peptide after glucagon stimulation test; eGFR = estimated glomerular filtration rate; ARNI = angiotensin receptor neprilysin inhibitor.

Although many reports have focused on the elevation of urinary C-peptide levels associated with sacubitril/valsartan, Ishibashi et al. indicated that serum C-peptide levels and CPI can be significantly elevated after sacubitril/valsartan administration, even in the absence of changes in IRI or glucose levels [13]. This suggests that neprilysin inhibition may lead to an overestimation of the insulin secretory capacity when assessed using static markers. Compared to IRI, the glucagon stimulation test-derived ΔCPR offers several advantages in evaluating endogenous insulin secretion. As previously noted, C-peptide is not present in exogenous insulin preparations and is more metabolically stable with a longer half-life and reduced variability [1]. Moreover, ΔCPR reflects stimulated insulin secretion with relatively low intra-individual variability and is less affected by transient factors such as stress or recent food intake [14]. These characteristics suggest that ΔCPR may serve as a more reliable and specific indicator of β-cell function, particularly in patients treated with sacubitril/valsartan, as the glucagon stimulation test directly assesses β-cell responsiveness to pharmacological stimulation and is less influenced by alterations in C-peptide metabolism. This may provide a more physiologically valid estimate of endogenous insulin secretion in the clinical context. However, a potential limitation of this approach should be noted. Neprilysin inhibition has been reported to impair glucagon degradation, leading to an increase in circulating glucagon level [15]. Although the clinical relevance of this effect remains uncertain, it may influence the results of glucagon stimulation tests in patients receiving sacubitril/valsartan. In the present case, while the impact of sacubitril/valsartan on ΔCPR is uncertain, the observed ΔCPR response appeared to be consistent with the actual clinical course. The patient was able to discontinue insulin therapy without deterioration in glycemic control, possibly indicating preservation of endogenous insulin secretion. The potential effects of neprilysin inhibition warrant further investigation.

Although the precise mechanism remains unclear, it is hypothesized that neprilysin inhibition by sacubitrilat may impair the renal degradation of C-peptide, leading to increased urinary excretion. In our literature review, although direct evidence that the C-peptide is a substrate of neprilysin was lacking [16, 17], it was noted that the C-peptide shares several structural features with known neprilysin substrates [16–18]. The persistently elevated U-CPR level after discontinuation may be explained by delayed recovery of neprilysin activity, possibly due to tissue-level accumulation of sacubitrilat or the preferential degradation of higher-affinity substrates during enzymatic recovery. However, further mechanistic studies are required to elucidate this interaction.

A limitation of our study was the lack of baseline U-CPR data prior to sacubitril/valsartan initiation. This restricted our ability to definitively attribute elevated U-CPR levels to the drug. Moreover, post-discontinuation insulin secretory capacity was not reassessed by dynamic testing, and this may have limited the interpretation of U-CPR changes over time.

In this case, U-CPR was observed to be disproportionately elevated despite normal endogenous insulin secretory capacity based on ΔCPR. This case provides insights into the interpretation of insulin secretion markers and may be employed in similar situations.

## Data Availability

No datasets were generated or analysed during the current study.

## Abbreviations

CPR: C-peptide immunoreactivity
CPI: C-peptide index
U-CPR: 24-h urinary C-peptide excretion rate
ΔCPR: Change in C-peptide immunoreactivity (serum CPR level before and after glucagon stimulation)
eGFR: Estimated glomerular filtration rate
MMT: Meal tolerance test
IRI: Immunoreactive insulin
ARNI: Angiotensin receptor neprilysin inhibitor
